# Latent Tuberculosis: Models, Computational Efforts and the Pathogen’s Regulatory Mechanisms during Dormancy

**DOI:** 10.3389/fbioe.2013.00004

**Published:** 2013-08-27

**Authors:** Gesham Magombedze, David Dowdy, Nicola Mulder

**Affiliations:** ^1^National Institute for Mathematical and Biological Synthesis, University of Tennessee, Knoxville, TN, USA; ^2^Johns Hopkins Bloomberg School of Public Health, Baltimore, MD, USA; ^3^Computational Biology Group, Department of Clinical Laboratory Sciences, Institute of Infectious Disease and Molecular Medicine, University of Cape Town, Cape Town, South Africa

**Keywords:** *Mycobacterium tuberculosis*, latency and dormancy regulation, latency models, mathematical and computational modeling

## Abstract

Latent tuberculosis is a clinical syndrome that occurs after an individual has been exposed to the *Mycobacterium tuberculosis* (Mtb) *Bacillus*, the infection has been established and an immune response has been generated to control the pathogen and force it into a quiescent state. Mtb can exit this quiescent state where it is unresponsive to treatment and elusive to the immune response, and enter a rapid replicating state, hence causing infection reactivation. It remains a gray area to understand how the pathogen causes a persistent infection and it is unclear whether the organism will be in a slow replicating state or a dormant non-replicating state. The ability of the pathogen to adapt to changing host immune response mechanisms, in which it is exposed to hypoxia, low pH, nitric oxide (NO), nutrient starvation, and several other anti-microbial effectors, is associated with a high metabolic plasticity that enables it to metabolize under these different conditions. Adaptive gene regulatory mechanisms are thought to coordinate how the pathogen changes their metabolic pathways through mechanisms that sense changes in oxygen tension and other stress factors, hence stimulating the pathogen to make necessary adjustments to ensure survival. Here, we review studies that give insights into latency/dormancy regulatory mechanisms that enable infection persistence and pathogen adaptation to different stress conditions. We highlight what mathematical and computational models can do and what they should do to enhance our current understanding of TB latency.

## Introduction

The pathogenesis of *Mycobacterium tuberculosis* (Mtb) is complex and involves an elaborate interaction with the host. Key factors, including the ability to survive in macrophages, the predilection for the lung, the formation of granulomas and long-term persistence, are poorly understood. The Mtb bacterial pathogen generally infects its mammalian host through the aerosol route. Inhalation of Mtb leads to phagocytosis by alveolar macrophages. After the onset of cell-mediated immune response, surviving bacteria are believed to enter a period of non-replicating persistence (NRP) in the phagosome until waning of host immunity results in reactivation from the latent state and the onset of disease. The ability of Mtb to persist for a long time in the latent state has been associated with the ability of the pathogen to maintain resistance against anti-microbial molecules and adaptation to host-induced metabolic constraints such as low nutrients, nitrogen, and oxygen stress (Wayne and Hayes, 1996; Betts et al., 2002; Hampshire et al., 2004; Voskuil et al., 2004b; Deb et al., 2009). In latency, Mtb are less metabolically active, and they have replication rates that are drastically diminished compared to bacilli in an active infection (Lillebaek et al., 2002, 2003). Tubercle bacilli require oxygen for growth; oxygen deprivation is lethal to them unless they have time to adapt to its gradual depletion. In the oxygen deprived environment if the bacteria are to survive, they need to alter their program of gene expression (Park et al., 2003; Schnappinger et al., 2003; Voskuil et al., 2003, 2004b; Rustad et al., 2008, 2009; Larsson et al., 2012), change their metabolic pathways, and depend on anaerobic respiration or to develop alternative mechanisms for generating energy. All these factors are potential contributors to latent TB infection (LTBI) development in humans.

The interaction between a pathogen and its host occurs on different scales. These range from molecular interactions, including the recognition of specific molecular patterns on innate immune cells by toll-like receptors, to interactions between individual cells, which, in turn, can range from the phagocytosis of bacteria by macrophages to the spread of disease through a host population and the emergence of different strains of pathogens in response to different host conditions. Current approaches to detection, prevention, and treatment of LTBI are inadequate, and rational development of new tools has been limited by poor understanding of the fundamental biology of LTBI, the *Bacillus*-host interaction, the immunologic parameters that are involved in establishing persistent infection, and the mechanisms of pathogen and host that lead to reactivation of Mtb and development of active disease.

LTBI represents a state of equilibrium in which the host shows no apparent symptoms, but is infected and where the immune system is only robust enough to contain the infection but is unable to clear it. When the immune system gets compromised, this balance is lost, leading to infection reactivation. From the host perspective, LTBI is clinically considered to be an asymptomatic, non-infectious state from which progression to active, infectious disease may occur at any time. Such progression occurs more often soon after the initial infection, and during times of host immune-suppression (e.g., human immunodeficiency virus infection, aging, therapy with tumor necrosis-alpha blockers) (Stead and Lofgren, 1983; Corbett et al., 2003; Gardam et al., 2003). Although classically considered to be a state in which Mtb is either contained or metabolically dormant, it is increasingly understood that clinical LTBI may represent a wide spectrum of host responses (Barry et al., 2009). Our lack of accurate and specific diagnostic tools to differentiate these responses *in vivo* makes the development of corresponding *in vitro* models similarly challenging.

## Global Prevalence of Latent TB

The global prevalence of LTBI is commonly cited as approximately 1 in 3, although no population-based evidence exists to verify this estimate. Such prevalence is roughly consistent with published population models of TB in globally representative settings (Abu-Raddad et al., 2009; Dowdy et al., 2013). The contribution of LTBI to population-level epidemiology differs widely by region; in high-incidence settings, recent infection contributes the majority of new cases of active TB, whereas in low-incidence settings, the majority of non-imported TB cases arise from reactivation of remote infection. Thus, while diagnosis and treatment of LTBI may substantially reduce the burden of active TB in settings of low or declining incidence (Comstock et al., 1979), it is unlikely to have similar population-level effects in high-burden areas until the ongoing annual risk of TB infection can be reduced. Nevertheless, as discussions regarding TB elimination garner increasing attention (Dye et al., 2012), it is becoming increasingly clear that this goal cannot be achieved without a strategy to diagnose and treat the massive reservoir of individuals with LTBI, in addition to those with active TB disease.

The lifetime risk of developing active TB after latent infection is often quoted as 10%, but modeling analyses suggest that, among individuals infected as adults, this number may be substantially higher (over 15%) (Vynnycky and Fine, 1997), and it is certainly much higher (up to 10% per year) among people living with untreated HIV (Corbett et al., 2003). The majority of this reactivation risk is experienced within the first 2 or 3 years following infection. Preventive therapy (traditionally with 6–9 months of daily isoniazid) can reduce this risk of reactivation by 60–70% (Smieja et al., 2000; Akolo et al., 2010) and is therefore a public health priority among individuals at high-risk of reactivation, including people living with HIV and people initiating biological therapy (e.g., TNF-alpha inhibitors) (Gardam et al., 2003) that affect the body’s ability to contain LTBI. However, in many parts of the world, such high-risk individuals comprise a relatively small proportion of the total population that will experience active TB from reactivation of latent infection. Plans for expanding access to preventive therapy among others with risk factors for reactivation (e.g., malnutrition, diabetes mellitus, adult household contacts) are therefore urgently needed and in most places lacking.

## Macrophages, Granuloma, Mtb Pathogen and LTBI Development

Macrophages are phagocytes at the frontline of host immune defense against microbial pathogens. They are the primary habitat of Mtb, and the pathogen preferentially targets macrophage vacuoles. Thus, the adaptation of Mtb to the phagosomal compartment of the macrophages is an essential component of its pathogenesis, transmission, and continual survival (Smeulders et al., 1999; Hampshire et al., 2004). This apparent incongruity demands that Mtb either tolerate the macrophage’s anti-microbial effectors, that is (i) low pH, (ii) reactive oxygen, and (iii) nitrogen species, or actively subvert normal cellular mechanisms to avoid being killed. It implies that for the pathogen to have extended survival in the host in a state of latency, the pathogen must somehow evade or interfere with the immune surveillance and signaling pathways (Voskuil et al., 2003, 2004b; Bacon et al., 2004; Berney and Cook, 2010; Taneja et al., 2010). Alternatively, the *Bacillus* must tolerate host defense mechanisms, either through mobilization of repair or detoxification pathways or through phenotypic tolerance developed as a result of metabolic adaptation or quiescence. After Mtb phagocytosis by macrophages, phagocytosed micro-organisms are rapidly transferred from phagosomes to lysosomes, where there is fusion of the phagosome with the late endosome and lysosome, and the microbes should be destroyed (Clemens and Horwitz, 1996; Fratti et al., 2001; Dubnau and Smith, 2003). The phagosomal environment is nitrosative, oxidative, functionally hypoxic, carbohydrate-poor, and capable of perturbing the pathogen’s envelope (Schnappinger et al., 2003). However, pathogenic mycobacteria resist lysosomal delivery and survive within macrophages inside mycobacterial phagosomes, in an intraphagosomal environment that is friendly to the bacterium. Macrophages, instead of killing the pathogen, have been shown to be manipulated by the bacilli, creating an environment suitable for intracellular replication by progressively translocating from phagolysosomes into the cytosol. The bacilli have been shown to be both anti-apoptotic, keeping the host cell alive to avoid the anti-microbial effects of apoptosis, and pro-necrotic, killing the host macrophage to allow infection of neighboring cells. This Mtb induced macrophage death was shown to be dependent on mycobacterial expression of ESAT-6 (van der Wel et al., 2007; Abdallah et al., 2011; Yu and Xie, 2012). Inhibition of Mtb phagosomal maturation was proposed as another mechanism for the survival of the pathogen in macrophages (Clemens and Horwitz, 1996; Fratti et al., 2001). Mtb is among the micro-organisms most successful at adapting to long-term residence in macrophage phagosomes. Phagosome arrest is a complex process and is not fully understood Taneja et al. (2010) (Clemens and Horwitz, 1996; Fratti et al., 2001, 2003; Anes et al., 2003). It is also speculated that pathogenic mycobacteria (including Mtb) might interfere directly in host trafficking pathways through expression of their own signal transduction molecules. The Mtb genome encodes two eukaryotic-like serine/threonine kinases [reviewed in Av-Gay and Everett (2000)] and protein kinase G (*PknG*), which consists of a kinase domain flanked by a large N- and C-terminal domain, and was shown to inhibit phagosome-lysosome fusion. Rapid lysosomal transfer of mycobacteria lacking *PknG* results in intracellular killing by bactericidal activities present in the lysosomes. Therefore, the ability of *PknG* to prevent lysosomal transfer suggests that it may affect survival of bacteria that have been internalized in macrophages (Walburger et al., 2004).

In latency, lipids have been found to be a major source of nutrients to the Mtb pathogen (McKinney, 2000; Ehrt and Schnappinger, 2007). Fatty acids have been hypothesized to constitute a significant source of both carbon and energy for non-replicating bacilli and the glyoxylate by-pass may serve in the formation of carbohydrates from fatty acids (Muñoz-Elías and McKinney, 2005; Ehrt and Schnappinger, 2007, 2009). Mtb strain (H37Rv) has a gene that encodes an isocitrate lyse, *icl1*, expressing ICL activity in macrophage infection. Studies by Graham and Clark-Curtiss (1999) and McKinney (2000) showed that a strain of Mtb in which the *icl* gene had been insertionally inactivated, lost the ability to persist in tissues of mice or in activated macrophages even though it did not appear to have diminished ability to survive *in vitro* anaerobiosis (McKinney, 2000). Isocitrate lyases catalyze an essential reaction of the glyoxylate shunt, an anaplerotic pathway that by-passes the CO2-generating steps of the tricarboxylic acid cycle, and enables bacteria to synthesize carbohydrates and replenish tricarboxylic acid cycle intermediates from fatty acid-derived acetyl-coenzyme A (Bishai, 2000; Ehrt and Schnappinger, 2007). For many bacteria, this pathway is required for growth on fatty acids as a sole carbon source. The genomes of most Mtb strains encode not one but two functional isocitrate lyases: *ICL1* and *ICL2*. Muñoz-Elías and McKinney (2005) showed that both *icl1*, *icl2* Mtb mutants can grow on different carbon sources (e.g., glycerol, glucose, acetate, propionate).

Another important facet of LTBI development is the granuloma. A granuloma is a multicellular structure comprised of macrophages (resting, activated, and infected), immune effector T cells on the periphery, chemokines, cytokines, adhesion molecules, and a caseous necrotic center (Lin et al., 2006; Lin and Flynn, 2010). Macrophages within a granuloma have two main functions (i) primarily to contain infection and (ii) for bacterial multiplication (Mtb preferred environment for growth). In the event that they fail to control infection, they end up harboring large amounts of bacteria and the bacteria can persist for decades, which is the case in LTBI. An impairment of the immune system will result in the disruption of the granuloma structure and collapse of the granuloma center, hence infection dissemination and potential transmission of the bacteria to other individuals. LTBI in humans comprise of a heterogeneous mixture of granulomas in both lungs and lymph nodes that provide a range of physiological micro-environments associated with bacterial replication and persistence, and therefore the basis for TB clinical latency (Barry et al., 2009).

## Mathematical Modeling and Latent Mtb Infection

Mathematical and computational efforts just like biological experiments have been key in unveiling many facets of infectious diseases. Their applications have immensely contributed to and shaped our current understanding of TB infection. In the same fashion as biological experiments, constructing, validating and analyzing mathematical, and computational models based on empirical observations can provide new insights into biological systems by making testable predictions and stir new directions for designing of biological experiments. Computational methods are our hope to handle and analyze vast amounts of high-throughput data. They are an essential tool in predictive studies of disease signatures, biomarkers, and vaccine and drug targets. Several mathematical schemes have been developed to provide a platform to study and understand host immune response mechanisms in active TB infection (Wigginton and Kirschner, 2001; Marino and Kirschner, 2004; Segovia-Juarez et al., 2004; Magombedze et al., 2006b, 2009, 2010; Sud et al., 2006; Ray et al., 2008, 2009). However, there is no substantial effort through mathematical modeling that has been put forth to enhance our understanding of LTBI (Magombedze and Mulder, 2011, 2012; Hegde et al., 2012; Magombedze et al., 2012).

In general, mathematical models have been useful in the evaluation, assessment, planning, and theoretical trial of vaccines and disease control intervention strategies of several infectious diseases and ailments (Scherer and McLean, 2002; Gandon and Day, 2007; Magombedze et al., 2008b; Smith and Schwartz, 2008; Heffernan and Keeling, 2009). The success or failure of a vaccine scheme can easily be predicted by use of such models when they are carefully developed and analyzed. This approach provides cost effective methods to give general insights and specific direction for implementing intervention programs (Long and Owens, 2011; Lugnér et al., 2012), hence providing direction for vaccinations. Application of optimal control and optimization techniques offers a platform to investigate and design optimal treatment, vaccine, and cost effective implementation protocols (Magombedze et al., 2011a,c). On the other hand, the effect of how these vaccines enhance the immune response in shrugging off the infection within the host has not been extensively studied compared to the extent of studies carried out at the population-level. Therapeutic and chemoprophylactic vaccines have been investigated in the studies (Koff et al., 2005; Smith and Schwartz, 2008; Johnson et al., 2011). Currently there is limited understanding for therapeutic vaccines and most of these vaccines offer partial immunity, which wanes over time. There has not been much new discovery for TB vaccines and BCG is still the vaccine that is recommended by WHO in infants. Several theories and debates exist as to what type of TB vaccine will be most effective, either an innate based (dendritic, macrophages, neutrophil) or a T cell based vaccine. A mathematical study (Magombedze et al., 2006a) was used to assess the efficacy of TB chemotherapy first line drug regimen following the directly observed therapy strategy (DOTs). This study was followed by models in which effects of treatment adherence and treatment when there is HIV coinfection were investigated (Magombedze et al., 2010, 2011b; Ramkissoon et al., 2012). This was designed to determine the possible treatment schemes when there are HIV and TB drug interactions, and several sequences of combinations of administering both HIV and TB drug cocktails, hence painting a better understanding of potential treatment schemes.

The current understanding of how the immune system interacts with the Mtb pathogen in TB infection owes credit to several mathematical studies (Wigginton and Kirschner, 2001; Marino and Kirschner, 2004; Magombedze et al., 2006b, 2008a, 2009; Sud et al., 2006; Ray et al., 2008, 2009; Day et al., 2009). However, the contribution of these studies toward the understanding of LTBI is still minimal. The first model of TB infection was in 1994 (Antia and Koella, 1994), which attempted to explain attributes of mycobacterial evolution and maintenance of micro-parasite virulence. Antia et al. (1996) developed another model on mycobacterial infections, where the mechanisms by which bacteria such as Mtb and *Mycobacterium leprae* persist at low densities for extended periods and attain high densities much later in the presence of an Ag specific immune response were investigated. Comprehensive and detailed modeling of Mtb was carried out in the studies (Wigginton and Kirschner, 2001; Marino and Kirschner, 2004; Segovia-Juarez et al., 2004; Magombedze et al., 2006b, 2008a, 2009, 2010; Sud et al., 2006), which described the interaction of the Mtb pathogen, macrophages, cytokines, and lymphocytes at the site of infection (lung compartment), and lung and lymph node compartments (Marino and Kirschner, 2004; Magombedze et al., 2009). These studies predicted attributes of the pathogen and immune response mechanisms that result in either LTBI or active TB, however without deciphering the mechanisms of LTBI development and persistence. Several other studies were carried, which showed the effects of TNF-alpha in granuloma formation and show how disruption of TNF-alpha mechanisms may enhance infection dissemination (Ray et al., 2008, 2009; Fallahi-Sichani et al., 2011). The mechanisms at the granuloma level that lead to different configurations of cells, however differential containment of the organisms are not well understood (Sharpe et al., 2009). There are current modeling efforts and methods for measuring and modulating TNF-alpha at the granuloma level to determine the effects on local control of Mtb infection using non-human primate data [reviewed in Kirschner et al. (2010)]. The studies (Ray et al., 2009; Fallahi-Sichani et al., 2011) present a way to study a single granuloma and the multi-scale molecular, cellular, and tissue events in the development of a granuloma, and show the critical role of TNF-alpha in control of bacterial growth in the granuloma.

There are studies that have started to incorporate details about signaling pathways into both immune cell and host-pathogen models, with the potential of identifying new drug targets (Goldstein et al., 2004; Franke et al., 2008; see review Kirschner and Linderman, 2009). Our study, Magombedze and Mulder (2011), is a first attempt to combine gene expression data, multiple stress factors, and bacterial cell densities using a mathematical frame work to investigate and explain several hypotheses on how persistence and reactivation of LTBI occurs. Results in this study echoes the results in the studies (Hegde et al., 2012; Magombedze and Mulder, 2012), were dynamics of several signaling and metabolic pathways mechanisms were explained using Boolean network modeling (Hegde et al., 2012), which used a bistable switch to help explain a potential mechanism on how the pathogen can switch between two different metabolic states. Our recent study (Magombedze and Mulder, 2012) predicted the regulation of the latency/dormancy program using a systems biology mathematical framework to be orchestrated by dynamic gene regulatory networks constituted of several DosR-regulon genes (Voskuil et al., 2003). This study predicted the role of DosR-regulon genes and other several genes as central in the regulation and maintenance of the latency program. In this study DosR-regulon genes were predicted to cluster with adaptation, detoxification, and virulence genes and several other genes of unknown or putative functions (Rv3131, Rv0569, Rv2032, Rv2530c, and Rv2694c), some of which were predicted both in the regulation of the stationary and non-replicating phase of the bacteria. This study furthermore predicted that other genes (Rv1133c, Rv2890c, Rv1177, Rv2710, Rv2532c, and Rv0982) to be also involved in the regulation of latency. These genes were shown in other studies (Sassetti and Rubin, 2003; Sassetti et al., 2003) to be essential for H37Rv growth and *in vivo* bacterial survival. Some of the genes predicted to be central in the regulation of latency are essential for bacterial growth (Rv3131), making them an attractive drug target. Other genes are known to have functions that are linked to human immune response mechanisms, such as induction of interferon-c (IFN-c) and interleukin-2 (IL-2) (Rv2032, Rv3133c, Rv3131, and Rv2031c), oxidative stress (Rv1909c), and nitrosative stress (Rv3131, Rv3133c, and Rv2031c). The latter are associated with nitrogen and oxygen reactive intermediate effector mechanisms of the immune response and were shown in other studies (Leyten et al., 2006; Roupie et al., 2007; Black et al., 2009) to have the potential to prime the human immune response. However, these predictions remain to be tested through biological experiments for potential LTBI drug design. With vast generation of data sets from different TB experimental infection models (mouse models, guinea pig, non-human primate models, and culture models) as well as data from real cases of infected people, mathematical and computational modeling (see Table 1 for current mathematical and computational methods) is our only hope to get sense of all data measured at different scales, through developing virtual models that can integrate data from different scales.

**Table 1:** **LTBI mathematical and computational models**.

LTBI mathematical and computational models (*in silico*)	Advantages	Disadvantages
Lung and lymph node models	Simplify complicated biological systems that are difficult to study experimentally in the lab	Results are dependent of model parameters and available data Complex to analyze
	Use computer simulation	
	Help to design new experiments and derive new hypotheses	
	Not expensive and flexible	
Granuloma models	Not expensive and flexible	Models the granuloma as an isolated structure
	Can be used to understand unknown mechanisms of the granuloma with computer simulations	Results are dependent on model assumptions and available data
Signaling and gene regulatory models	Can predict gene regulatory mechanisms in Mtb latency/dormancy adaptation	Depends on available data and the modeling approach used
	Easy to simulated with a computer	Difficult to develop and analyze
Multi-scale models	Link different scales: organs, tissues, cells, and molecules	Difficult to develop, analyze, and simulate
	Model latency in a more realistic way	They are complicated
Computational methods (models)	Ability to analyze data from different kinds of experiments	Computationally intensive
	Offer a platform to integrate data and mathematical models	Depends on available data
	Predictive tools for identifying vaccine and drug candidates	Involves a combination of different sophisticated mathematical, statistical, and programing techniques

## Understanding Latent Mtb and Identifying New Drug Targets through Computational Efforts

Several computational models and bioinformatics techniques have revealed network interactions, metabolic, and signaling pathways associated with the development of the latent infection; and with prediction of gene functional groups, hence providing informative data sets for future experiments (Mazandu and Mulder, 2011a,b; Mazandu et al., 2011; Hegde et al., 2012; Magombedze and Mulder, 2012). Mazandu and Mulder (2011a) used a combination of sequence and functional genomics data to generate a functional interaction network for Mtb, which was used for drug target prediction and function prediction for the proteins of unknown function (Mazandu and Mulder, 2011b,c). The data integration method exploited sequence similarity and shared domains, gene synteny, phylogenetic profiles, co-occurrence in abstracts, co-expression and other protein–protein interaction prediction techniques to predict functional interactions between all possible protein pairs in the Mtb proteome. A combined score was attributed to each interaction based on the individual scores from each evidence type. Using only medium to high confidence interactions, a network of 4,136 proteins and 58,000 interactions was generated, and was found to have a small world property, which is common among biological networks. This network was used to predict functions for uncharacterized proteins in Mtb, which constitute a high percentage of the genome. Most of the uncharacterized proteins were found to have metabolic activities, but others were predicted to be involved in transcription regulation, signal transduction, and pathogenesis, suggesting that there are many currently uncharacterized proteins that could play a role in the survival and persistence of Mtb during infection and reacting to changes in the environment (Mazandu and Mulder, 2011b,c).

Functional interaction networks provide the advantage of enabling a systems level view of an organism. Proteins are not viewed in isolation, and for each protein we can establish its relative importance in the biological system by assessing its network properties. Network properties include degree (number of direct interacting partners), betweenness, closeness, and other measures of how well connected a protein is within the network and whether its removal will have a substantial effect on the rest of the network. Like mathematical modeling where we can model expression or metabolic changes and determine the effect of “knocking out” a gene or protein, in biological networks we can knock out proteins and determine the effect on the network. Mazandu and Mulder (2011a) used their Mtb functional interaction network to identify possible drug targets by ranking proteins by their betweenness and centrality measures. Using this technique, they identified 881 possible drug targets, whose elimination would affect a number of other proteins and interactions. These proteins included 114 previously identified drug targets in either UniProtKB or TDR targets (http://tdrtargets.org), as well as targets for some of the currently used anti-TB drugs (Mazandu and Mulder, 2011a). Zheng et al. (2012) used protein–protein interactions networks from STRING for identifying virulence proteins in several pathogens, including Mtb. Using the interaction network, they identified virulence factors based on number of neighbors and strength of interactions and compared this to a feature selection method and BLAST approaches. Their results were benchmarked against a database of validated virulence factors and the network-based method was found to out-perform the other two methods (Zheng et al., 2012).

These network studies, which are purely computational, allow us to predict effects of perturbations on the whole biological system and can help us to identify genes or proteins that known dormancy genes interact with. For example, the DosR protein has over 75 interactions with other proteins in the DosR-regulon, as well as with other regulators and proteins whose functions are not yet known. The host-pathogen interactions, which link two functional interaction networks, allow us to then determine how these proteins potentially interact with host proteins. Computational tools have also been used to identify new vaccine candidates for TB. For example, there are some new possible vaccine candidates on the horizon, such as the latency antigens α-crystallin (HspX) and Rv2660c of the DosR-regulon, which were identified from studies of gene expression changes during dormancy (Wang et al., 2013). In addition, Gideon et al. (2012), using a bioinformatics and empirical approach, identified several novel TB proteins that elicited an anti-tuberculosis T cell response.

## Mtb Latency and Dormancy Models

The bacterial behavior during the growth phase (lag, log, stationary, and death phases) is well known, but little is known about how they behave in LTBI. The ability of the pathogen to enter and exit from different states has associated it with its ability to cause persistent infection. In this state it is not clearly known whether it will be in a persistent slow replicating state or a dormant non-replicating state, thus ultimately causing a latent infection with the potential to reactivate to active disease. The terminology for latent TB state and dormant bacterial state is somehow confusing and is used differently in different studies. TB Latency refers to an *in vivo* situation where the bacteria and the host have established a balanced state without causing apparent symptoms. Whereas the term dormant refers to the physiological and reversible metabolic shutdown state of the bacteria, which is normally simulated in *in vitro* culture models.

*In vitro* models have been used to model latency or investigate the adaptive processes of the Mtb to non-proliferating conditions which can be achieved by limiting at least one of the essential conditions required for Mtb growth (for a summary of the latency/dormancy models see Table 2). These include: (i) starvation of essential nutrients, such as carbon, nitrogen, or phosphorus and (ii) depletion of oxygen, which prevents aerobic respiration by the obligate aerobe. The adaptation to oxygen is the most widely studied of these conditions. Low-oxygen levels have long been recognized to limit Mtb replication while promoting long-term survival, which is associated with a distinct physiologic adaptation and marked by bacteriostasis in addition to metabolic, chromosomal, and structural changes of the bacilli (Wayne and Hayes, 1996; Wayne and Sohaskey, 2001). These states can be distinguished using a resuscitation reculture failure test (Sun and Zhang, 1999). Resuscitation of non-colony forming cultures only confirms the difference between dead cells and dormant cells. Truly dormant cells were shown to resume replication after stimulation with a resuscitation promoting factor (*Rpf*), a 16-kDa protein derived from a Gram-positive coccus *Micrococcus luteus* (*M. luteus*) [reviewed in Zhang (2004)]. The studies (Mukamolova et al., 2002; Tufariello et al., 2004; Downing et al., 2005; Shleeva et al., 2010) showed over-expression of five of the Mtb *Rpf* genes that could stimulate the growth of tubercle bacilli from an old culture with a small inoculum. It may be concluded that the *Rpf* proteins are directly involved in reactivation of persisting bacteria. However, the mechanism of the activating effect of *Rpf* on dormant cells is still not completely understood.

**Table 2:** **Latency/dormancy *in vitro* and *in vivo* models**.

Latency/dormancy model	Advantages	Disadvantages
Wayne model (*in vitro*, culture)	Can simulate different Mtb bacteria physiological dormant states	Uses a single stress factor (hypoxia)
	Inexpensive and easy to carry out	Does not reflect what truly happens *in vivo*
	Easy for expressed genes profiling	Slow simulation of dormancy
Rapid anaerobic model (*in vitro*, culture)	Inexpensive and easy to carry out	Uses a single stress factor (hypoxia)
	Rapid simulation of Mtb dormant state	Difficult to correlate with *in vivo* latency
	Easy for expressed genes profiling	
Multi-stress model (*in vitro*, culture)	Easily achieve the stationary and non-replicating phases of Mtb	Difficult to correlate with *in vivo* latency
	Not expensive and easy to carry out	
	Easy for expressed genes profiling	
Cornell model (*in vivo*, mouse model)	Inexpensive and easy to handle	Latency development does not compare well to human LTBI
	Availability of genetic variant strains	
	Large number of immunological tools and reagents	
	Can simulate latency/dormancy	
Guinea pig/rabbit model (*in vivo*)	Easy to handle	Limited availability of reagents
	Show necrosis and granuloma structure similar to humans	Lack of true latency which resembles human LTBI
Non-human primate model (*in vivo*)	Similar immunological and infection pathology with humans	Expensive
	Develop LTBI similar to humans	Require trained personnel (veterinary scientists) to handle
	Availability of reagents	Ethical issues

### The Wayne model

Wayne and Hayes (1996), established an “*in vivo* model for dormancy” where *in vitro* growth TB cultures were subjected to gradual oxygen depletion to mimic tubercle bacilli *in vivo*. A sealed, standing culture is allowed to incubate over a period of days while the bacteria deplete the available oxygen. The culture becomes progressively more hypoxic with a concomitant shift in Mtb physiology (Wayne, 1977; Wayne and Lin, 1982). Gentle stirring and a defined culture-to-headspin ratio improve reproducibility (Wayne and Hayes, 1996). Two distinct states of NRP that reflect discrete metabolic and drug susceptibility states compared with log phase growth are illustrated. The first stage, designated NRP 1, occurred when the declining oxygen level reached 1% saturation. This stage is characterized by increased production of glycine dehydrogenase and steady ATP generation. The second stage, NRP 2, occurred when the oxygen level reached 0.06% saturation and this stage is characterized by a marked decline of glycine dehydrogenase and susceptibility to metronidazole. The dormant cells in the Wayne model remained responsive to heat shock and were culturable when transferred to fresh aerated medium (Shleeva et al., 2004, 2010). Therefore, it is thought that the Wayne model probably represents the initial stage of formation of a dormant state and essentially reflects bacterial adaptation to low-oxygen level rather than to the dormant state. A study by Shleeva et al. (2002) showed that longer incubation time of several months in the Wayne model can produce some dormant bacilli.

### The rapid anaerobic model

There are several other studies (Honaker et al., 2009; Leistikow et al., 2010) that have improved and modified the Wayne model to yield consistent and more precise results. In these studies, a rapid anaerobic dormancy model is implemented. This model differs from the Wayne model in the larger size of stir bars and faster rate of stirring resulting in a more homogeneous population of bacilli, and even oxygen distribution throughout the culture. The model exhausts available oxygen rapidly ensuring achievement of anaerobiosis in a relatively short time interval compared to the Wayne model.

### Cornell model

The most convincing evidence of dormant Mtb was demonstrated by McCune Jr. et al. (1956, 1966) in a mouse model (Zhang, 2004; Shleeva et al., 2010). Mice were infected with virulent Mtb and the infection was allowed to establish for 2 weeks, followed by treatment with a combination of INH and PZA for 3 months. Initially no bacilli could be detected in the mouse spleen; however, one third of the mice relapsed with culture-positive tubercle bacilli when the treatment was discontinued for 3 months (McCune Jr. et al., 1956; Hu et al., 2000) and more relapses were noticed after administration of immunosuppressive steroids (McCune Jr. et al., 1966). This was an indication that dormant bacilli insensitive to antibiotic treatment persisted. There are variations of the Cornell model that show the same disappearance and reappearance phenomenon of tubercle bacilli in mice with different infectious dose, route of infection (aerosol or intravenous), and different drug combinations (Scanga et al., 1999).

### Multi-stress model

The limitation with single stress models, like hypoxia, nutrient starvation, and exposure to NO is that they cannot closely imitate the *in vivo* latency/dormancy stimulating conditions. Unfortunately there is no substantive repertoire of alternative models that can model latency with multiple stresses. To date, the study of Deb et al. (2009) is the only one that has achieved this. This multiple stress model used a combination of low-oxygen (5%), high CO2 (10%), low nutrients (10% Dubos medium), and an acidic pH of 5.0. This study illustrates how the Mtb pathogen adapts and prepares for the dormancy stage. Under these conditions the pathogen was shown to lose its acid-fastness characteristics, while at the same time it accumulated wax esters and lipids, a process thought to be an energy storing strategy as it shifts from aerobic respiration to anaerobic respiration. Several studies (Hampshire et al., 2004; Deb et al., 2009; Berney and Cook, 2010) have suggested that, continual survival of Mtb in the anaerobic respiration state is sustained through energy generation from metabolism of fatty acids. This model revealed high expression of genes involved in the glyoxylate cycle and energy metabolism, which is a strong indication that the pathogen had switched to anaerobic respiration (Deb et al., 2009; Patel et al., 2011).

### Non-human primate model

There are several *in vivo* models that are currently used to experimentally explain the development of LTBI. These include, (i) mouse models (for example the Cornell model), (ii) the Guinea pig/rabbit model, with the potential to simulate granulomas similar to those observed in humans. Unfortunately it is difficult to observe LTBI manifestation in Guinea pigs, which are highly susceptible to rapid disease progression proceeding low-dose aerosol infection. Although the pathology in lungs of Guinea pigs and rabbits is reminiscent of human pathology, there is relative lack of immunologic reagents for these animals (Flynn et al., 2003). The most attractive model to date is the non-human primate model. Non-human primates (*Cynomolgus macaque*) have physiological, infection pathology, immunological similarities with humans, and they make it possible to carry out experiments that are difficult or impossible to perform in the human population. Its ability to generate clinical correlates of infection has made the non-human primate model important in the study of LTBI. The downside of this model includes cost, difficulty with handling animals, containment of biohazards, and ethical considerations.

## Latency and Dormancy Regulation

Survival of Mtb bacilli *in vivo* is attributed to the pathogen’s ability to adapt to the changing environment. This is orchestrated by a program of genes that sense changes of stress factors (hypoxia, NO-exposure, heat shock, nutrient starvation, pH change, and low iron) in its environment. The phagosome environment is associated with production of oxidative bursts, the synthesis of inducible nitric oxide (NO) synthase resulting in the production of NO and other reactive nitrogen intermediates, which are responsible for the killing of intracellular bacteria. This changing environment within the macrophages should elicit new programs of gene expression in the pathogen if it is to survive. The Mtb pathogens possess mechanisms to detect the ambient oxygen tension based on the *DosT/DosS/DosR* system and WhiB3 sensory systems, which enable them to adapt to changes in oxygen availability by adjusting their metabolism accordingly (Park et al., 2003; Kumar et al., 2007; Rustad et al., 2008; Honaker et al., 2009).

The *DosS/DosT* and *DosR* response regulator is a two component system, which consists of a sensor kinase (component 1) which autophosphorylates histidine, then transphosphorylates a conserved aspartate of its cognate transcriptional regulator (component 2). Mtb has eleven complete systems and seven orphans belonging to sensor kinase and response regulator families (Cole et al., 1998). *DosR* has been shown to act as a transcriptional regulator, which is responsible for the induction of nearly all hypoxia-induced genes of Mtb (Park et al., 2003; Voskuil et al., 2003, 2004b; Kumar et al., 2007; Rustad et al., 2008). It has been shown that, recombinant sensor kinases *DosS* and *DosT* autophosphorylate to stable phosphoproteins (Malhotra et al., 2006; Murphy and Brown, 2007), but rapidly ransphosphorylate *DosR* under conditions of low-oxygen tension or in the presence of NO or CO (Voskuil et al., 2003; Kumar et al., 2007; Sousa et al., 2007; Shiloh et al., 2008), confirming these as members of the two component family. *DosS* is a redox sensor and *DosT* is a hypoxia sensor. Aerobically, *DosS* is in its inactive Fe3+ form, as a result of its rapid oxidation by O2. When *DosS* is reduced to Fe2+, its autokinase activity is increased and uses this oxidation state to signal *DosR* activation (Honaker et al., 2009). *DosT* exists aerobically in the O2-bound form, which is inactive and its resistance to oxidation removes the possibility of its functioning as a redox sensor. The study of Honaker et al. (2009) shows that the *DosR-*regulon is induced in stepwise manner with *DosT* only important in early stages and *DosS* important in the late stages. This is because *DosT* is not able to signal *DosR* expression efficiently under low-oxygen. The early activity by *DosT* is thought to kick start induction of *DosR-*regulon genes, among them *DosS*. As the *DosR-*regulon is expressed, levels of *DosS* become elevated, enabling further induction of the regulon.

The ability of the Mtb bacteria to adapt to changing oxygen concentration has been linked with the role of the *WhiB3* protein implicated in sensing oxygen tension and redox state by mycobacteria. The *WhiB3* gene was found to be strongly induced in Mtb during acute infection of mouse lungs and also during growth in resting bone marrow-derived macrophages, but repressed after IFN-gamma activation of the macrophages (Banaiee et al., 2006). The study of Singh et al. (2007) was able to show that WhiB3 contains a 4Fe–4S cluster, which can bind NO. In the presence of oxygen, the WhiB3 [4Fe–4S]2+ cluster is degraded first to a [3Fe–4S]+ cluster, then to [2Fe–2S]2+, and subsequently lost altogether (Crack et al., 2004; Singh et al., 2007; Alam and Agrawal, 2008). WhiB3-mediated response to the presence of oxygen, therefore, is predicted to occur through direct control of the activity of metabolic proteins or through modification of transcriptional regulators (Alam and Agrawal, 2008). A role for WhiB3 in regulation of the transcriptional machinery in mycobacteria may be supported by the finding that WhiB3 interacts with the major sigma factor, *SigA* (*RpoV*) (Steyn et al., 2002), but the effect of this interaction on *SigA* activity is not known, and further studies are required to understand the precise role of WhiB3 in mediating any adaptation of mycobacteria to changes in oxygen tension.

### The DosR-regulon

The DosR-regulon is a significant regulator of genes in response to hypoxia and NO [see Table 3 for the list of *DosR-*regulon genes (Park et al., 2003; Kumar et al., 2008)]. This, combined with the production of NO by host immune cells and the likely hypoxic nature of the granuloma, makes a strong prediction that this regulon is a primary trigger of metabolic shift down to achieve the non-replicative state. Latency and dormancy models (Park et al., 2003; Voskuil et al., 2003, 2004b; Honaker et al., 2009) have predicted that LTBI is regulated through the *DosR*-regulon/system. Roberts et al. (2004) showed that deletion of *DosS* or *DosT* in the H37Rv strain causes a reduced expression of hypoxia reporter gene and complete failure of *hspX* stimulation in the double mutant. Studies (Voskuil et al., 2003; Kumar et al., 2007; Sousa et al., 2007; Shiloh et al., 2008) predicted that without the NO signal, the DosR-regulon will not be up-regulated, and cell division will continue. The *DosR-*regulon was shown to be weakly induced in the stationary phase indicating partial stimulation of the dormancy genes. With increased oxygen tension there is increased expression of the transcripts *fdxA*, *pfkB*, *narX*, and *narK2*, which indicate a switch to anaerobic metabolism from aerobic respiration (Bacon et al., 2004; Voskuil et al., 2004b). These genes were also observed to be induced in NO exposure and nutrient starvation (Betts et al., 2002; Bacon et al., 2004; Hampshire et al., 2004; Deb et al., 2009). Dormancy genes expressed in the stationary phase and the NRP were shown (Voskuil et al., 2003, 2004b) to be located in “operon like clusters.” One such cluster of genes (*rsbW*, *Rv3288c*, *Rv3289c*, and *lat*) is seen in the study Hampshire et al. (2004) and has previously been identified as up-regulated in the stationary phase survival study by Betts et al. (2002). Other gene clusters include *Rv0982-Rv0984 (moaB2)*, *Rv1460-Rv1463*, *Rv3138-Rv3141* (*pflA*, *fadE24*, *fadE23*, and *fadB4*, respectively), and *Rv3458c-Rv3460c*. The largest cluster of genes includes *Rv3864*, *Rv3867-Rv3871*, *Rv3876*, and *Rv3878*, the majority of which fall within the RD-1 region deleted from strain BCG Pasteur. A complete list of genes up-regulated and down regulated in hypoxia, NO exposure, nutrient starvation, and other stress conditions are found in the studies (Betts et al., 2002; Park et al., 2003; Voskuil et al., 2003, 2004b; Hampshire et al., 2004; Deb et al., 2009).

**Table 3:** **Mtb DosR-regulon genes**.

Rv number	Gene name	Protein function (probable)	Rv number	Gene name	Protein function (probable)
Rv0079		HP	Rv2028c		Universal stress protein
Rv0080		CHP	Rv2029c	*pfkB*	Phosphofructokinase
Rv0081		Transcriptional factor	Rv2030c		CHP
Rv0082		Oxidoreductase	Rv2031c	*hspX*	Heat shock protein
Rv0083		Oxidoreductase	Rv2032	*Acg*	CHP
Rv0569		CHP	Rv2623	*TB31.7*	Universal stress protein
Rv0570	*nrdZ*	Ribonucleotide red.	Rv2624c		Universal stress protein
Rv0571c		CHP	Rv2625c		Leucine rich protein
Rv0572c		HP	Rv2626c	*hrp1*	Hypoxic response protein
Rv0574c		CHP	Rv2627c		CHP
MT0639		HP	Rv2628		HP
Rv1733c		Transmembrane protein	Rv2629		CHP
Rv1734c		CHP	Rv2630		HP
Rv1736c	*narX*	Nitrate reductase	Rv2631		CHP
Rv1737c	*narK2*	Nitrite transporter	Rv2830c	*vapB22*	Antitoxin VapB22
Rv1738		CHP	Rv3126c		HP
Rv1812c		Dehydrogenase	Rv3127		CHP
Rv1813c		CHP	Rv3128c		CHP
Rv1996		Universal stress protein	Rv3129		CHP
Rv1997	*ctpF*	Cation trans. ATPase	Rv3130c	*tgs1*	Triacylglycerol synthase
Rv2003c		CHP	Rv3131		CHP
Rv2004c		HP	Rv3132c	*dosS*	Sensor hist. Kinase
Rv2005c		Universal stress protein	Rv3133c	*dosR*	Two-comp. resp. reg.
Rv2006	*otsB1*	Trehalose phosphatize	Rv3134c		Universal stress protein
Rv2007c	*fdxA*	Ferredoxin	Rv3841	*bfrB*	Bacterioferritin
Rv2027c	*dosT*	Sensor hist. kinase			

*A list of dosR-regulon genes. dosT is part of the dosR-regulon but it is not up-regulated in response to many conditions tested to date. HP, hypothetical protein; CHP, conserved hypothetical protein*.

### Latency/dormancy stationary phase operons

Using hierarchical clustering Voskuil et al. (2004b) showed that most stationary phase-specific induced genes are transcriptionally clustered and thus share a common regulatory profile. These genes include *Rv0106*, *PPE3*, *Rv1535*, *PE20*, *PPE31*, *rpsR2*, *rpsN2*, *rpmG1*, and *rpmB2*. The four ribosome encoding genes are separated from the majority of ribosome encoding genes on the Mtb genome, perhaps indicating a difference in function. By contrast, most ribosomal protein genes are repressed during stationary phase as well as in NRP. It is thought that, the ribosomal proteins encoded by *rpsR2*, *rpsN2*, *rpmG1*, and *rpmB2* are involved in increasing ribosomal fidelity during non-proliferating conditions. The *desA3* gene (encoding a probable fatty acid desaturase) is highly induced in the transition to stationary phase, but not during NRP. Interestingly, the other two fatty acid desaturase genes found in the Mtb genome are induced early during NRP (Bacon et al., 2004; Voskuil et al., 2004b). Also, the *desA* genes are induced, with *desA3* being strongly repressed throughout NRP, while *desA1* and *desA2* are only induced during stationary phase. The fact that all three genes are induced during the transition to non-proliferating conditions while the bacilli are still replicating indicates the bacilli may be changing the degree of saturation of the cell wall mycolic acids in preparation for a non-proliferating state. Genes of the *PE* and *PPE* families (which constitute 8% of the genes in the Mtb genome) are also differentially regulated between stationary phase and NRP. *PE3*, *PE20*, and *PPE31* are induced during stationary phase, while *PE11*, *PE34*, *PE-PGRS11*, *PE-PGRS14*, *PPE17*, *PPE47*, and *PPE48* are induced during NRP, and many PE/PPE genes are repressed under both conditions (Voskuil et al., 2004a). There is speculation on the functions of these proteins that involve their ability to provide antigenic variation and interfere with immune responses, in addition to performing a purely structural role.

In addition to the dormancy regulon, several other genes are strongly up-regulated in the NRP model. Most of these genes are repressed late in the NRP model, with the exception of *hsp*, *Rv0678*, *Rv0841c*, *Rv1734c*, *Rv1874*, *mez*, and *Rv2660c* (Voskuil et al., 2003, 2004b). Interestingly, *Rv1734c* is a dormancy regulon gene that is weakly induced early in NRP but is the only dormancy gene that was noticed to remain expressed in the late stages of NRP. The continued induction of *Rv1734c*, and the induction of a few other dormancy genes, when most dormancy genes are no longer induced, indicates there may be another level of control for some of the dormancy genes other than through activation by *DosR*. Two strongly induced genes early in NRP (*ahpC* and *ahpD*) encode the alkyl hydroperoxide reductase and are strongly repressed throughout the adaptation to stationary phase. *AhpC* is a peroxidase and peroxynitrate reductase is involved in antioxidant defense mechanisms in Mtb. Genes encoding cytochrome *bd* oxidase (*cydA*, *cydB*, *cydC*, and *cydD*) are also expressed early in the NRP model. The cytochrome *bd-1* oxidase is an alternative terminal oxidase for the aerobic respiratory chain that is believed to function at low-oxygen levels due to its higher affinity for oxygen than the primary cytochrome *c* oxidase. Therefore, the induction of these genes is consistent with the depletion of oxygen early in the NRP model (Voskuil et al., 2003, 2004b; Hampshire et al., 2004). Induction of the *bfrB* gene that encodes the iron storage protein bacterioferritin, *bfrB* is repressed when the mycobactin synthesis genes are induced under low iron conditions. The iron-dependent regulation of *bfrB* and the mycobactin genes require the presence of *IdeR*. The fact that the mycobactin and *bfrB* genes are co-induced indicates that *bfrB* can be regulated by a mechanism independent of the IdeR-dependent activation that is observed during conditions of low iron. During NRP, the bacilli may attempt to increase iron stores for use during long periods of dormancy. Therefore, Mtb appears to induce both uptake and storage functions simultaneously.

Transcriptome analysis of stationary phase Mtb cells incubated under standard conditions (Betts et al., 2002; Voskuil et al., 2004b; Shleeva et al., 2010) revealed activation of some of the DosR-regulon genes, including those encoding the two component regulatory system of stress response, as well as nitrate reductase, nitrite binding protein, and alpha crystalline. It may therefore be suggested that expression of the DosR-regulon not only acts as a marker of a hypoxic state, but is also responsible for development of the universal cellular stress response. However, transcriptome analysis of Mtb under oxygen limitation revealed that enhanced expression of the DosR operon genes under hypoxia was brief and occurred only at the initial stage. A bigger group of genes was then activated, reflecting the “true” cellular response to hypoxia (Rustad et al., 2008; Shleeva et al., 2010).

### Latency and Mtb adaptation to changes in pH

The macrophage intracellular environment is associated with pH changes that are detrimental to bacterial survival, as such, a change in expression of a large number of Mtb genes over a range of pH values is noticed (Rao et al., 2001; Fisher et al., 2002; Saviola et al., 2003). This implies that mycobacteria are able to perceive the pH of their environment. Two adenylyl cyclases have been suggested to fulfill that role in Mtb. *Rv1264* was observed to have an increased fold change of adenylyl cyclase activity at pH 6 compared to pH 8, mediated through a pH-responsive catalytic domain (Tews et al., 2005). In response to pH, the protein undergoes conformational rearrangements and these may involve an unsaturated fatty acid as a structural element (Tews et al., 2005; Findeisen et al., 2007). In the closely related adenylyl cyclase *Rv2212*, pH-sensing activity is strongly induced by binding of unsaturated fatty acids, but the mechanism of this effect is not fully understood (Motaal et al., 2006). While both *Rv1264* and *Rv2212* have increased activity at acidic pH, the mechanism for activation was reported to be different between the two proteins (Tews et al., 2005; Motaal et al., 2006).

### Adaptation to nutrient depletion

A rapid reduction in the ability of bacteria to synthesize proteins leads to the accumulation of the stringent response signaling molecule *(p)ppGpp* which in turn reduces transcription of stable RNA genes (Dahl et al., 2003; Jain et al., 2006). The enzyme *relAb* (the only *ppGpp* synthase homolog in Mtb) is required for survival of mycobacteria under long-term *in vitro* starvation conditions. Microarray studies characterizing the response of Mtb to both a fall in available nutrients and hypoxia, have demonstrated a rise in *relA* (Betts et al., 2002; Hampshire et al., 2004). The study of Hampshire et al., 2004, showed that *relA* is involved in initial nutrient depletion adaptation in facilitating long-term survival (Primm et al., 2000; Chatterji and Kumar Ojha, 2001). The type of regulatory pattern seen for *relA* was also observed for *aceAb*. It has previously been suggested that increasing levels of *(p)ppGpp* in *E. coli* may be the sole inducer of *qor*, a gene shown to be up-regulated in response to conditions of stress, which seems to function by reducing the quinone pool (Chang et al., 2002; Dürrschmid et al., 2008). However, in the study of Hampshire et al. (2004), following the drop in expression of *relA* early on, *qor* stayed up-regulated throughout the time course and even showed a late substantial increase, suggesting that other levels of regulation are present.

Different types of stress tend to up-regulate different sigma factors, however *sigB*, *sigE*, and *sigH* are a subset of sigma factors associated with the entire period of stationary phase adaptation (Betts et al., 2002; Hampshire et al., 2004). Alternative sigma factors are a major category of transcriptional regulators responsible for orchestrating the gene expression responses to non-proliferating conditions in *B. subtilis* and *E. coli*. The sigma factor genes *sigF*, *sigB*, and *sigJ* are the ones that are induced significantly during stationary phase [reviewed in Wang et al. (2011)]. Voskuil et al. (2004b) showed that only *sigB* is induced during stationary phase, while *sigF* and *sigJ* are expressed relative to their levels in exponentially growing bacilli and there is a weak induction of *sigE*. *SigC* is induced early, while *sigH* is induced late in the NRP model. The study (Voskuil et al., 2004b) spells out the need to determine if the state of Mtb during clinical latency more closely resembles the *E. coli* Sigma S model or the *Bacillus* sporulation cascade paradigm.

The genes involved in Mtb persistence *in vivo* are believed to fall into two groups: those responsible for transition to the persisting state and those responsible for its maintenance (Betts et al., 2002; Shleeva et al., 2010). For example, the Mtb mutant lacking the *relA* gene (its product in *ppGpp*), a “strong response” mediator, regulating cellular activity (Dahl et al., 2003) was capable of normal growth in the macrophages and *in vitro* with citrate and phospholipids as carbon sources, but did not survive transition into the stationary phase or incubation under anaerobic conditions (Primm et al., 2000; Shleeva et al., 2010). The expression of a number of genes in the mutant strain and in the wild type was different, including the genes encoding the reactivating protein: *rpfA* (enhanced expression) and *rpfC* (inhibited expression). This demonstrates that the *relA* product is required for persistence under starvation, oxygen limitation, and in an extensive stationary phase, as well as in the chronic model and in the artificial granuloma in mice *in vivo*; it is one of the candidates for participation in the persistent state in humans.

## Concluding Remarks

Understanding the development and persistence of LTBI infection is complex. Despite many years of research and several strides that have been made in identifying key Mtb persistence factors, gene signatures for vaccine and drug design, eradication of TB still seems to be out of reach. The main short coming of current studies is the inadequacy of the current *in vivo* and *in vitro* latency models [reviewed in Patel et al. (2011)] to closely imitate latency development in humans. Nevertheless, these *in vitro* single stress models have shaped our current understanding on bacilli adaptation to the stationary and non-replicating stages. This has revealed key functional groups of genes that regulate latency and dormancy development. Most of these models are not close to modeling the real human latency development, which in humans can persistent for the entire life span until an opportune moment for reactivation, since they are designed to simulate latency/dormancy over a few hours or days. Development of models that follow the framework proposed in the study Deb et al. (2009), in which several stress factors are considered, has the potential to illuminate and enhance our current understanding of Mtb persistence. The use of the non-human primate model is more encouraging since it simulates latency/dormancy close to that of humans (Flynn et al., 2003), and has become the most attractive model to study LTBI compared to other models. However, it is marred with genotype variability and small sample sizes which affects model results reproducibility. The complexity associated with handling this model makes it expensive and unmanageable for several scientists.

Several studies reviewed here suggest that under each different stress condition the bacteria has a unique set of genes that sense and report changes in its environment, hence facilitating necessary changes for adaptation. These mechanisms seem to be dynamic with the length of time spent in each condition as well as the type of the stress. However, overlaps of genes expressed in hypoxia, NO-exposure, and nutrient starvation are noticed (Betts et al., 2002; Voskuil et al., 2003, 2004b; Hampshire et al., 2004). These kinds of experiments generate lots of data and this is where we think mathematical and computational modeling should play a crucial role. Studies (Magombedze and Mulder, 2011, 2012; Mazandu and Mulder, 2011a,b,c; Hegde et al., 2012), show how computational and mathematical methods can be used to analyze, learn more, identify traits and properties of protein–protein interactions, gene regulatory mechanisms, and metabolic pathways that are active in latency development and maintenance, which cannot be identified by carrying out basic statistical analysis of the experimental data. A few strides have been made so far to achieve this goal. The study (Fallahi-Sichani et al., 2011) applied a multi-scale approach to study the granuloma using a granuloma model with multiple single cell entities modeled separately to understand TNF trafficking within the granuloma and how this correlates to control of infection. The studies (Magombedze and Mulder, 2011, 2012) are the only recent studies to attempt to model gene cross talk and regulation that occurs in latency adaptation and interface change in gene expression with Mtb cell populations to show how these gene regulations influence the final physiological and metabolic stages of the pathogen. The future should see development of more computational schemes that integrate data from different scales in an endeavor to understand the dynamics of LTBI. A multi-scale model that captures measurements of cells (T cells), cytokines (influence of TNF-alpha and IFN-gamma), signaling mechanisms of immune cells when there is an infection, pathogen mechanisms of interfering with the immune signaling pathways, pathogen gene regulatory mechanisms and how these mechanisms influence the pathogen phenotypic characteristics and metabolic pathways into one framework will be a good basis to generate and test several hypotheses and has potential to usher the study of LTBI on to a new level.

Existing models of the immune system do not cover much detail at the molecular level such as protein sequences and structures and the effect of polymorphisms. The use of bioinformatics is resourceful in this respect, it allows for analysis of pathogen genome sequences and prediction of protein–protein interactions within the pathogen, and between the pathogen and host (Leung and Cavalieri, 2003; Kendall et al., 2004). Availability of the genome sequence helps in compilation of all the potential gene products encoded by a particular organism, identification of functions that are missing or unique in a particular organism and identifying genes that are common between prokaryotes and eukaryotes. This enhances host-pathogen interaction studies which could provide important clues for development of more rational and specific methods to search for new drug targets, vaccine candidates and diagnostic tests.

## Conflict of Interest Statement

The authors declare that the research was conducted in the absence of any commercial or financial relationships that could be construed as a potential conflict of interest.

## References

[B1] Abdallah A. M. Bestebroer J. Savage N. D. de Punder K. van Zon M. Wilson L. (2011). Mycobacterial secretion systems ESX-1 and ESX-5 play distinct roles in host cell death and inflammasome activation. J. Immunol. 187, 4744–4753 10.4049/jimmunol.1101457 21957139

[B2] Abu-Raddad L. J. Sabatelli L. Achterberg J. T. Sugimoto J. D. Longini I. M. Jr. Dye C. (2009). Epidemiological benefits of more-effective tuberculosis vaccines, drugs, and diagnostics. Proc. Natl. Acad. Sci. U.S.A. 106, 13980–13985 10.1073/pnas.0901720106 19666590 PMC2720405

[B3] Akolo C. Adetifa I. Shepperd S. Volmink J. (2010). Treatment of latent tuberculosis infection in HIV infected persons. Cochrane Database Syst. Rev. 1, CD000171 20091503 10.1002/14651858.CD000171.pub3 PMC7043303

[B4] Alam S. Agrawal P. (2008). Matrix-assisted refolding and redox properties of WhiB3/Rv3416 of *Mycobacterium tuberculosis* H37Rv. Protein Expr. Purif. 61, 83–91 10.1016/j.pep.2008.04.010 18550384

[B5] Anes E. Kühnel M. P. Bos E. Moniz-Pereira J. Habermann A. Griffiths G. (2003). Selected lipids activate phagosome actin assembly and maturation resulting in killing of pathogenic mycobacteria. Nat. Cell Biol. 5, 793–802 10.1038/ncb1036 12942085

[B6] Antia R. Koella J. C. (1994). A model of non-specific immunity. J. Theor. Biol. 168, 141–150 10.1006/jtbi.1994.1094 8022194

[B7] Antia R. Koella J. C. Perrot V. (1996). Models of the within-host dynamics of persistent mycobacterial infections. Proc. R. Soc. Lond. B Biol. Sci. 263, 257–263 10.1098/rspb.1996.0040 8920248

[B8] Av-Gay Y. Everett M. (2000). The eukaryotic-like Ser/Thr protein kinases of *Mycobacterium tuberculosis*. Trends Microbiol. 8, 238–244 10.1016/S0966-842X(00)01734-0 10785641

[B9] Bacon J. James B. W. Wernisch L. Williams A. Morley K. A. Hatch G. J. (2004). The influence of reduced oxygen availability on pathogenicity and gene expression in *Mycobacterium tuberculosis*. Tuberculosis 84, 205–217 10.1016/j.tube.2003.12.011 15207490

[B10] Banaiee N. Jacobs W. Jr. Ernst J. (2006). Regulation of *Mycobacterium tuberculosis* whiB3 in the mouse lung and macrophages. Infect. Immun. 74, 6449–6457 10.1128/IAI.00190-06 16923787 PMC1695489

[B11] Barry C. E. Boshoff H. I. Dartois V. Dick T. Ehrt S. Flynn J. (2009). The spectrum of latent tuberculosis: rethinking the biology and intervention strategies. Nat. Rev. Microbiol. 7, 845–855 10.1038/nrmicro2236 19855401 PMC4144869

[B12] Berney M. Cook G. M. (2010). Unique flexibility in energy metabolism allows mycobacteria to combat starvation and hypoxia. PLoS ONE 5:e8614 10.1371/journal.pone.0008614 20062806 PMC2799521

[B13] Betts J. C. Lukey P. T. Robb L. C. McAdam R. A. Duncan K. (2002). Evaluation of a nutrient starvation model of *Mycobacterium tuberculosis* persistence by gene and protein expression profiling. Mol. Microbiol. 43, 717–731 10.1046/j.1365-2958.2002.02779.x 11929527

[B14] Bishai W. (2000). Microbiology: lipid lunch for persistent pathogen. Nature 406, 683–685 10.1038/35021159 10963578

[B15] Black G. F. Thiel B. A. Ota M. O. Parida S. K. Adegbola R. Boom W. H. (2009). Immunogenicity of novel DosR regulon-encoded candidate antigens of *Mycobacterium tuberculosis* in three high-burden populations in Africa. Clin. Vaccine Immunol. 16, 1203–1212 10.1128/CVI.00111-09 19553548 PMC2725533

[B16] Chang D.-E. Smalley D. J. Conway T. (2002). Gene expression profiling of *Escherichia coli* growth transitions: an expanded stringent response model. Mol. Microbiol. 45, 289–306 10.1046/j.1365-2958.2002.03001.x 12123445

[B17] Chatterji D. Kumar Ojha A. (2001). Revisiting the stringent response, ppGpp and starvation signaling. Curr. Opin. Microbiol. 4, 160–165 10.1016/S1369-5274(00)00182-X 11282471

[B18] Clemens D. L. Horwitz M. A. (1996). The *Mycobacterium tuberculosis* phagosome interacts with early endosomes and is accessible to exogenously administered transferrin. J. Exp. Med. 184, 1349–1355 10.1084/jem.184.4.1349 8879207 PMC2192850

[B19] Cole S. Brosch R. Parkhill J. Garnier T. Churcher C. Harris D. (1998). Deciphering the biology of *Mycobacterium tuberculosis* from the complete genome sequence. Nature 393, 537–544 10.1038/31159 9634230

[B20] Comstock G. W. Baum C. Snider D. E. Jr. (1979). Isoniazid prophylaxis among Alaskan Eskimos: a final report of the bethel isoniazid studies. Am. Rev. Respir. Dis. 119, 827–830 453704 10.1164/arrd.1979.119.5.827

[B21] Corbett E. L. Watt C. J. Walker N. Maher D. Williams B. G. Raviglione M. C. (2003). The growing burden of tuberculosis: global trends and interactions with the HIV epidemic. Arch. Intern. Med. 163, 1009–1021 10.1001/archinte.163.9.1009 12742798

[B22] Crack J. Green J. Thomson A. J. (2004). Mechanism of oxygen sensing by the bacterial transcription factor fumarate-nitrate reduction (FNR). J. Biol. Chem. 279, 9278–9286 10.1074/jbc.M309878200 14645253

[B23] Dahl J. L. Kraus C. N. Boshoff H. I. Doan B. Foley K. Avarbock D. (2003). The role of RelMtb-mediated adaptation to stationary phase in long-term persistence of *Mycobacterium tuberculosis* in mice. Proc. Natl. Acad. Sci. U.S.A. 100, 10026–10031 10.1073/pnas.1631248100 12897239 PMC187750

[B24] Day J. Friedman A. Schlesinger L. S. (2009). Modeling the immune rheostat of macrophages in the lung in response to infection. Proc. Natl. Acad. Sci. U.S.A. 106, 11246–11251 10.1073/pnas.0904846106 19549875 PMC2708732

[B25] Deb C. Lee C.-M. Dubey V. S. Daniel J. Abomoelak B. Sirakova T. D. (2009). A novel in vitro multiple-stress dormancy model for *Mycobacterium tuberculosis* generates a lipid-loaded, drug-tolerant, dormant pathogen. PLoS ONE 4:e6077 10.1371/journal.pone.0006077 19562030 PMC2698117

[B26] Dowdy D. W. Basu S. Andrews J. R. (2013). Is passive diagnosis enough?: the impact of subclinical disease on diagnostic strategies for tuberculosis. Am. J. Respir. Crit. Care Med. 187, 543–551 10.1164/rccm.201207-1217OC 23262515 PMC3733406

[B27] Downing K. J. Mischenko V. V. Shleeva M. O. Young D. I. Young M. Kaprelyants A. S. (2005). Mutants of *Mycobacterium tuberculosis* lacking three of the five rpf-like genes are defective for growth in vivo and for resuscitation in vitro. Infect. Immun. 73, 3038–3043 10.1128/IAI.73.5.3038-3043.2005 15845511 PMC1087353

[B28] Dubnau E. Smith I. (2003). *Mycobacterium tuberculosis* gene expression in macrophages. Microbes Infect. 5, 629–637 10.1016/S1286-4579(03)00090-X 12787739

[B29] Dürrschmid K. Reischer H. Schmidt-Heck W. Hrebicek T. Guthke R. Rizzi A. (2008). Monitoring of transcriptome and proteome profiles to investigate the cellular response of *E. coli* towards recombinant protein expression under defined chemostat conditions. J. Biotechnol. 135, 34–44 10.1016/j.jbiotec.2008.02.013 18405993

[B30] Dye C. Glaziou P. Floyd K. Raviglione M. (2012). Prospects for tuberculosis elimination. Annu. Rev. Public Health 34, 271–286 10.1146/annurev-publhealth-031912-114431 23244049

[B31] Ehrt S. Schnappinger D. (2007). *Mycobacterium tuberculosis* virulence: lipids inside and out. Nat. Med. 13, 284–285 10.1038/nm0307-284 17342139

[B32] Ehrt S. Schnappinger D. (2009). Mycobacterial survival strategies in the phagosome: defence against host stresses. Cell. Microbiol. 11, 1170–1178 10.1111/j.1462-5822.2009.01335.x 19438516 PMC3170014

[B33] Fallahi-Sichani M. El-Kebir M. Marino S. Kirschner D. E. Linderman J. J. (2011). Multiscale computational modeling reveals a critical role for TNF-α receptor 1 dynamics in tuberculosis granuloma formation. J. Immunol. 186, 3472–3483 10.4049/jimmunol.1003299 21321109 PMC3127549

[B34] Findeisen F. Linder J. U. Schultz A. Schultz J. E. Brügger B. Wieland F. (2007). The structure of the regulatory domain of the adenylyl cyclase Rv1264 from *Mycobacterium tuberculosis* with bound oleic acid. J. Mol. Biol. 369, 1282–1295 10.1016/j.jmb.2007.04.013 17482646

[B35] Fisher M. A. Plikaytis B. B. Shinnick T. M. (2002). Microarray analysis of the *Mycobacterium tuberculosis* transcriptional response to the acidic conditions found in phagosomes. J. Bacteriol. 184, 4025–4032 10.1128/JB.184.14.4025-4032.2002 12081975 PMC135184

[B36] Flynn J. L. Capuano S. V. Croix D. Pawar S. Myers A. Zinovik A. (2003). Non-human primates: a model for tuberculosis research. Tuberculosis (Edinb) 83, 116–118 10.1016/S1472-9792(02)00059-8 12758200

[B37] Franke R. Müller M. Wundrack N. Gilles E.-D. Klamt S. Kähne T. (2008). Host-pathogen systems biology: logical modelling of hepatocyte growth factor and *Helicobacter pylori* induced c-Met signal transduction. BMC Syst. Biol. 2:4 10.1186/1752-0509-2-4 18194572 PMC2254585

[B38] Fratti R. A. Backer J. M. Gruenberg J. Corvera S. Deretic V. (2001). Role of phosphatidylinositol 3-kinase and Rab5 effectors in phagosomal biogenesis and mycobacterial phagosome maturation arrest. J. Cell Biol. 154, 631–644 10.1083/jcb.200106049 11489920 PMC2196432

[B39] Fratti R. A. Chua J. Vergne I. Deretic V. (2003). *Mycobacterium tuberculosis* glycosylated phosphatidylinositol causes phagosome maturation arrest. Proc. Natl. Acad. Sci. U.S.A. 100, 5437–5442 10.1073/pnas.0737613100 12702770 PMC154363

[B40] Gandon S. Day T. (2007). The evolutionary epidemiology of vaccination. J. R. Soc. Interface 4, 803–817 10.1098/rsif.2006.0207 17264055 PMC2394540

[B41] Gardam M. A. Keystone E. C. Menzies R. Manners S. Skamene E. Long R. (2003). Anti-tumour necrosis factor agents and tuberculosis risk: mechanisms of action and clinical management. Lancet Infect. Dis. 3, 148–155 10.1016/S1473-3099(03)00545-0 12614731

[B42] Gideon H. P. Wilkinson K. A. Rustad T. R. Oni T. Guio H. Sherman D. R. (2012). Bioinformatic and empirical analysis of novel hypoxia-inducible targets of the human antituberculosis T cell response. J. Immunol. 189, 5867–5876 10.4049/jimmunol.1202281 23169589 PMC3519923

[B43] Goldstein B. Faeder J. R. Hlavacek W. S. (2004). Mathematical and computational models of immune-receptor signalling. Nat. Rev. Immunol. 4, 445–456 10.1038/nri1374 15173833

[B44] Graham J. E. Clark-Curtiss J. E. (1999). Identification of *Mycobacterium tuberculosis* RNAs synthesized in response to phagocytosis by human macrophages by selective capture of transcribed sequences (SCOTS). Proc. Natl. Acad. Sci. U.S.A. 96, 11554–11559 10.1073/pnas.96.20.11554 10500215 PMC18072

[B45] Hampshire T. Soneji S. Bacon J. James B. W. Hinds J. Laing K. (2004). Stationary phase gene expression of *Mycobacterium tuberculosis* following a progressive nutrient depletion: a model for persistent organisms? Tuberculosis (Edinb) 84, 228 10.1016/j.tube.2003.12.010 15207492 PMC3195342

[B46] Heffernan J. Keeling M. J. (2009). Implications of vaccination and waning immunity. Proc. R. Soc. Lond. B Biol. Sci. 276, 2071–2080 10.1098/rspb.2009.0057 19324753 PMC2677258

[B47] Hegde S. R. Rajasingh H. Das C. Mande S. S. Mande S. C. (2012). Understanding communication signals during mycobacterial latency through predicted genome-wide protein interactions and Boolean modeling. PLoS ONE 7:e33893 10.1371/journal.pone.0033893 22448278 PMC3309013

[B48] Honaker R. W. Leistikow R. L. I. Bartek L. Voskuil M. I. (2009). Unique roles of DosT and DosS in DosR regulon induction and *Mycobacterium tuberculosis* dormancy. Infect. Immun. 77, 3258–3263 10.1128/IAI.01449-08 19487478 PMC2715697

[B49] Hu Y. Mangan J. A. Dhillon J. Sole K. M. Mitchison D. A. Butcher P. D. (2000). Detection of mRNA transcripts and active transcription in persistent *Mycobacterium tuberculosis* induced by exposure to rifampin or pyrazinamide. J. Bacteriol. 182, 6358–6365 10.1128/JB.182.22.6358-6365.2000 PMC94781 11053379

[B50] Jain V. Kumar M. Chatterji D. (2006). ppGpp: stringent response and survival. J. Microbiol. 44, 1 16554711

[B51] Johnson P. L. Kochin B. F. McAfee M. S. Stromnes M. Regoes R. R. Ahmed R. (2011). Vaccination alters the balance between protective immunity, exhaustion, escape, and death in chronic infections. J. Virol. 85, 5565–5570 10.1128/JVI.00166-11 21411537 PMC3094965

[B52] Kendall S. L. Rison S. C. Movahedzadeh F. Frita R. Stoker N. G. (2004). What do microarrays really tell us about *M. tuberculosis*? Trends Microbiol. 12, 537–544 10.1016/j.tim.2004.10.005 15539113

[B53] Kirschner D. E. Linderman J. J. (2009). Mathematical and computational approaches can complement experimental studies of host–pathogen interactions. Cell. Microbiol. 11, 531–539 10.1111/j.1462-5822.2008.01281.x 19134115 PMC2720090

[B54] Kirschner D. E. Young D. Flynn J. L. (2010). Tuberculosis: global approaches to a global disease. Curr. Opin. Biotechnol. 21, 524–531 10.1016/j.copbio.2010.06.002 20637596 PMC2943033

[B55] Koff W. C. Johnson P. R. Watkins D. I. Burton D. R. Lifson J. D. Hasenkrug K. J. (2005). HIV vaccine design: insights from live attenuated SIV vaccines. Nat. Immunol. 7, 19–23 10.1038/ni1296 16357854

[B56] Kumar A. Deshane J. S. Crossman D. K. Bolisetty S. Yan B.-S. Kramnik I. (2008). Heme oxygenase-1-derived carbon monoxide induces the *Mycobacterium tuberculosis* dormancy regulon. J. Biol. Chem. 283, 18032–18039 10.1074/jbc.M802274200 18400743 PMC2440631

[B57] Kumar A. Toledo J. C. Patel R. P. Lancaster J. R. Steyn A. J. C. (2007). *Mycobacterium tuberculosis* DosS is a redox sensor and DosT is a hypoxia sensor. Proc. Natl. Acad. Sci. U.S.A. 104, 11568–11573 10.1073/pnas.0705054104 17609369 PMC1906723

[B58] Larsson C. Luna B. Ammerman N. C. Maiga M. Agarwal N. Bishai W. R. (2012). Gene expression of *Mycobacterium tuberculosis* putative transcription factors whiB1-7 in redox environments. PLoS ONE 7:e37516 10.1371/journal.pone.0037516 22829866 PMC3400605

[B59] Leistikow R. L. Morton R. A. Bartek I. L. Frimpong I. Wagner K. Voskuil M. I. (2010). The *Mycobacterium tuberculosis* DosR regulon assists in metabolic homeostasis and enables rapid recovery from nonrespiring dormancy. J. Bacteriol. 192, 1662–1670 10.1128/JB.00926-09 20023019 PMC2832541

[B60] Leung Y. F. Cavalieri D. (2003). Fundamentals of cDNA microarray data analysis. Trends Genet. 19, 649–659 10.1016/j.tig.2003.09.015 14585617

[B61] Leyten E. Lin M. Y. Franken K. Friggen A. H. Prins C. Van Meijgaarden K. E. (2006). Human T-cell responses to 25 novel antigens encoded by genes of the dormancy regulon of *Mycobacterium tuberculosis*. Microbes Infect. 8, 2052 10.1016/j.micinf.2006.03.018 16931093

[B62] Lillebaek T. Dirksen A. Baess I. Strunge B. Thomsen V. Ø Andersen ÅB. (2002). Molecular evidence of endogenous reactivation of *Mycobacterium tuberculosis* after 33 years of latent infection. J. Infect. Dis. 185, 401–404 10.1086/338342 11807725

[B63] Lillebaek T. Dirksen A. Vynnycky E. Baess I. Thomsen V. Ø Andersen ÅB. (2003). Stability of DNA patterns and evidence of *Mycobacterium tuberculosis* reactivation occurring decades after the initial infection. J. Infect. Dis. 188, 1032–1039 10.1086/378240 14513424

[B64] Lin P. L. Flynn J. L. (2010). Understanding latent tuberculosis: a moving target. J. Immunol. 185, 15–22 10.4049/jimmunol.0903856 20562268 PMC3311959

[B65] Lin P. L. Pawar S. Myers A. Pegu A. Fuhrman C. Reinhart T. A. (2006). Early events in *Mycobacterium tuberculosis* infection in cynomolgus macaques. Infect. Immun. 74, 3790–3803 10.1128/IAI.74.4.2052-2062.2006 16790751 PMC1489679

[B66] Long E. F. Owens D. K. (2011). The cost-effectiveness of a modestly effective HIV vaccine in the United States. Vaccine 29, 6113–6124 10.1016/j.vaccine.2011.04.013 21510996 PMC3156325

[B67] Lugnér A. K. van Boven M. de Vries R. Postma M. J. Wallinga J. (2012). Cost effectiveness of vaccination against pandemic influenza in European countries: mathematical modelling analysis. BMJ 345:e4445 10.1136/bmj.e4445 22791791 PMC3395306

[B68] Magombedze G. Chiyaka C. Mukandavire Z. (2011a). Optimal control of malaria chemotherapy. Nonlinear Anal. Modell. Contr. 16, 415–434

[B69] Magombedze G. Garira W. Mwenje E. Bhunu C. (2011b). “*Mycobacterium tuberculosis* treatment and the emergence of a multi-drug resistant strain in the lungs,” in Infectious Disease Modelling Research Progress Series: Public Health in the 21st Century, eds Tchuenche J. M. Chayaka C. (New York: Nova Publishers), 197–227

[B70] Magombedze G. Garira W. Mwenje E. Bhunu C. P. (2011c). Optimal control for HIV-1 multi-drug therapy. Int. J. Comput. Math. 88, 314–340

[B71] Magombedze G. Dube N. Mulder N. (2012). “Mathematical modeling of infection and treatment of *Mycobacterium tuberculosis* latency,” in A Treatise of Biological Models. Series: Mathematics Research Developments, eds Nyabadza F. Kgosimore M. Lungu E. (New York: Nova Publishers), Chap. 2, 9–34

[B72] Magombedze G. Garira W. Mwenje E. (2006a). Mathematical modeling of chemotherapy of human TB infection. J. Biol. Syst. 14, 509–553 10.1142/S0218339006001945

[B73] Magombedze G. Garira W. Mwenje E. (2006b). Modelling the human immune response mechanisms to *Mycobacterium tuberculosis* infection in the lungs. Math. Biosci. Eng. 3, 661 10.3934/mbe.2006.3.661 20361838

[B74] Magombedze G. Garira W. Mwenje E. (2008a). In-vivo mathematical study of co-infection dynamics of HIV-1 and *Mycobacterium tuberculosis*. J. Biol. Syst. 16, 357–394 10.1142/S0218339008002551

[B75] Magombedze G. Garira W. Mwenje E. (2008b). Modelling the immunopathogenesis of HIV-1 infection and the effect of multi-drug therapy: the role of fusion inhibitors in HAART. Math. Biosci. Eng. 5, 485–504 10.3934/mbe.2008.5.485 18616354

[B76] Magombedze G. Garira W. Mwenje E. (2009). The role of dendritic cells and other immune mechanisms during human infection with *Mycobacterium tuberculosis*. Int. J. Biomath. 2, 69–105 10.1142/S1793524509000534

[B77] Magombedze G. Garira W. Mwenje E. (2010). Modeling the TB/HIV-1 co-infection and the effects of its treatment. Math. Popul. Stud. 17, 12–64 10.1080/08898480903467241

[B78] Magombedze G. Mulder N. (2011). A mathematical representation of the development of *Mycobacterium tuberculosis* active, latent and dormant stages. J. Theor. Biol. 292, 44–59 10.1016/j.jtbi.2011.09.025 21968442

[B79] Magombedze G. Mulder N. (2012). Understanding TB latency using computational and dynamic modelling procedures. Infect. Genet. Evol. 13, 267–283 10.1016/j.meegid.2012.09.017 23146828

[B80] Malhotra V. Sharma D. Ramanathan V. Shakila H. Saini D. K. Chakravorty S. (2006). Disruption of response regulator gene, devR, leads to attenuation in virulence of *Mycobacterium tuberculosis*. FEMS Microbiol. Lett. 231, 237–245 10.1016/S0378-1097(04)00002-3 14987770

[B81] Marino S. Kirschner D. E. (2004). The human immune response to *Mycobacterium tuberculosis* in lung and lymph node. J. Theor. Biol. 227, 463–486 10.1016/j.jtbi.2003.11.023 15038983

[B82] Mazandu G. K. Mulder N. J. (2011a). Generation and analysis of large-scale data-driven *Mycobacterium tuberculosis* functional networks for drug target identification. Adv. Bioinformatics 2011 10.1155/2011/801478 22190924 PMC3235424

[B83] Mazandu G. K. Mulder N. J. (2011b). Scoring protein relationships in functional interaction networks predicted from sequence data. PLoS ONE 6:e18607 10.1371/journal.pone.0018607 21526183 PMC3079720

[B84] Mazandu G. K. Mulder N. J. (2011c). Using the underlying biological organization of the *Mycobacterium tuberculosis* functional network for protein function prediction. Infect. Genet. Evol. 12, 922–932 10.1016/j.meegid.2011.10.027 22085822

[B85] Mazandu G. K. Opap K. Mulder N. J. (2011). Contribution of microarray data to the advancement of knowledge on the *Mycobacterium tuberculosis* interactome: use of the random partial least squares approach. Infect. Genet. Evol. 11, 725–733 10.1016/j.meegid.2010.09.003 21514402

[B86] McCune R. M. Jr. Tompsett R. McDermott W. (1956). The fate of *Mycobacterium tuberculosis* in mouse tissues as determined by the microbial enumeration technique II. The conversion of tuberculous infection to the latent state by the administration of pyrazinamide and a companion drug. J. Exp. Med. 104, 763–802 10.1084/jem.104.5.737 13367342 PMC2136612

[B87] McCune R. M. Feldmann F. M. Lambert H. P. McDermott W. (1966). Microbial persistence I. The capacity of tubercle bacilli to survive sterilization in mouse tissues. J. Exp. Med. 123, 445–468 10.1084/jem.123.3.445 4957010 PMC2138153

[B88] McKinney J. D. (2000). In vivo veritas: the search for TB drug targets goes live. Nat. Med. 6, 1330–1333 10.1038/82142 11100116

[B89] Motaal A. A. Tews I. Schultz J. E. Linder J. U. (2006). Fatty acid regulation of adenylyl cyclase Rv2212 from *Mycobacterium tuberculosis* H37Rv. FEBS J. 273, 4219–4228 10.1111/j.1742-4658.2006.05420.x 16925585

[B90] Mukamolova G. V. Turapov O. A. Kazarian K. Telkov M. Kaprelyants A. S. Kell D. B. (2002). The rpf gene of *Micrococcus luteus* encodes an essential secreted growth factor. Mol. Microbiol. 46, 611–621 10.1046/j.1365-2958.2002.03184.x 12410820

[B91] Muñoz-Elías E. J. McKinney J. D. (2005). *Mycobacterium tuberculosis* isocitrate lyases 1 and 2 are jointly required for in vivo growth and virulence. Nat. Med. 11, 638–644 10.1038/nm1252 15895072 PMC1464426

[B92] Murphy D. Brown J. (2007). Identification of gene targets against dormant phase *Mycobacterium tuberculosis* infections. BMC Infect. Dis. 7:84 10.1186/1471-2334-7-84 17655757 PMC1950094

[B93] Park H.-D. Guinn K. M. Harrell M. I. Liao R. Voskuil M. I. Tompa M. (2003). Rv3133c/dosR is a transcription factor that mediates the hypoxic response of *Mycobacterium tuberculosis*. Mol. Microbiol. 48, 833–843 10.1046/j.1365-2958.2003.03474.x 12694625 PMC1992516

[B94] Patel K. Jhamb S. S. Singh P. P. (2011). Models of latent tuberculosis: their salient features, limitations, and development. J. Lab. Physicians 3, 75 10.4103/0974-2727.86837 22219558 PMC3249721

[B95] Primm T. P. Andersen S. J. Mizrahi V. Avarbock D. Rubin H. Barry C. E. (2000). The stringent response of *Mycobacterium tuberculosis* is required for long-term survival. J. Bacteriol. 182, 4889–4898 10.1128/JB.182.17.4889-4898.2000 10940033 PMC111369

[B96] Ramkissoon S. Mwambi H. G. Matthews A. P. (2012). Modelling HIV and MTB co-infection including combined treatment strategies. PLoS ONE 7:e49492 10.1371/journal.pone.0049492 23209581 PMC3509125

[B97] Rao M. Streur T. L. Aldwell F. E. Cook G. M. (2001). Intracellular pH regulation by *Mycobacterium smegmatis* and *Mycobacterium bovis* BCG. Microbiology 147, 1017–1024 11283297 10.1099/00221287-147-4-1017

[B98] Ray J. C. Flynn J. L. Kirschner D. E. (2009). Synergy between individual TNF-dependent functions determines granuloma performance for controlling *Mycobacterium tuberculosis* infection. J. Immunol. 182, 3706–3717 10.4049/jimmunol.0802297 19265149 PMC3182770

[B99] Ray J. C. Wang J. Chan J. Kirschner D. E. (2008). The timing of TNF and IFN-gamma signaling affects macrophage activation strategies during *Mycobacterium tuberculosis* infection. J. Theor. Biol. 252, 24–38 10.1016/j.jtbi.2008.01.010 18321531 PMC2459258

[B100] Roberts D. M. Liao R. P. Wisedchaisri G. Hol W. G. Sherman D. R. (2004). Two sensor kinases contribute to the hypoxic response of *Mycobacterium tuberculosis*. J. Biol. Chem. 279, 23082–23087 10.1074/jbc.M401230200 15033981 PMC1458500

[B101] Roupie V. Romano M. Zhang L. Korf H. Lin M. Y. Franken K. L. (2007). Immunogenicity of eight dormancy regulon-encoded proteins of *Mycobacterium tuberculosis* in DNA-vaccinated and tuberculosis-infected mice. Infect. Immun. 75, 941–949 10.1128/IAI.01137-06 17145953 PMC1828490

[B102] Rustad T. R. Harrell M. I. Liao R. Sherman D. R. (2008). The enduring hypoxic response of *Mycobacterium tuberculosis*. PLoS ONE 3:e1502 10.1371/journal.pone.0001502 18231589 PMC2198943

[B103] Rustad T. R. Sherrid A. M. Minch K. J. Sherman D. R. (2009). Hypoxia: a window into *Mycobacterium tuberculosis* latency. Cell. Microbiol. 11, 1151–1159 10.1111/j.1462-5822.2009.01325.x 19388905

[B104] Sassetti C. M. Boyd D. H. Rubin E. J. (2003). Genes required for mycobacterial growth defined by high density mutagenesis. Mol. Microbiol. 48, 77–84 10.1046/j.1365-2958.2003.03425.x 12657046

[B105] Sassetti C. M. Rubin E. J. (2003). Genetic requirements for mycobacterial survival during infection. Proc. Natl. Acad. Sci. U.S.A. 100, 12989–12994 10.1073/pnas.2134250100 14569030 PMC240732

[B106] Saviola B. Woolwine S. C. Bishai W. R. (2003). Isolation of acid-inducible genes of *Mycobacterium tuberculosis* with the use of recombinase-based in vivo expression technology. Infect. Immun. 71, 1379–1388 10.1128/IAI.71.3.1379-1388.2003 12595455 PMC148880

[B107] Scanga C. A. Mohan V. Joseph H. Yu K. Chan J. Flynn J. L. (1999). Reactivation of latent tuberculosis: variations on the Cornell murine model. Infect. Immun. 67, 4531–4538 10456896 10.1128/iai.67.9.4531-4538.1999 PMC96774

[B108] Scherer A. McLean A. (2002). Mathematical models of vaccination. Br. Med. Bull. 62, 187–199 10.1093/bmb/62.1.187 12176860

[B109] Schnappinger D. Ehrt S. Voskuil M. I. Liu Y. Mangan J. A. Monahan M. (2003). Transcriptional adaptation of *Mycobacterium tuberculosis* within macrophages insights into the phagosomal environment. J. Exp. Med. 198, 693–704 10.1084/jem.20030846 12953091 PMC2194186

[B110] Segovia-Juarez J. L. Ganguli S. Kirschner D. (2004). Identifying control mechanisms of granuloma formation during *M. tuberculosis* infection using an agent-based model. J. Theor. Biol. 231, 357–376 10.1016/j.jtbi.2004.06.031 15501468

[B111] Sharpe S. Eschelbach E. Basaraba R. Gleeson F. Hall G. McIntyre A. (2009). Determination of lesion volume by MRI and stereology in a macaque model of tuberculosis. Tuberculosis 89, 405–416 10.1016/j.tube.2009.09.002 19879805

[B112] Shiloh M. U. Manzanillo P. Cox J. S. (2008). *Mycobacterium tuberculosis* senses host-derived carbon monoxide during macrophage infection. Cell Host Microbe 3, 323–330 18474359 10.1016/j.chom.2008.03.007 PMC2873178

[B113] Shleeva M. Bagramyan K. Telkov M. Mukamolova G. V. Young M. Kell D. B. (2002). Formation and resuscitation of ‘non-culturable’cells of Rhodococcus rhodochrous and *Mycobacterium tuberculosis* in prolonged stationary phase. Microbiology 148, 1581–1591 11988533 10.1099/00221287-148-5-1581

[B114] Shleeva M. Mukamolova G. V. Young M. Williams H. D. Kaprelyants A. S. (2004). Formation of ‘non-culturable’cells of *Mycobacterium smegmatis* in stationary phase in response to growth under suboptimal conditions and their Rpf-mediated resuscitation. Microbiology 150, 1687–1697 10.1099/mic.0.26893-0 15184555

[B115] Shleeva M. Salina E. Kaprelyants A. (2010). Dormant forms of mycobacteria. Microbiology 79, 1–12 10.1134/S0026261710010017

[B116] Singh A. Guidry L. Narasimhulu K. Mai D. Trombley J. Redding K. E. (2007). *Mycobacterium tuberculosis* WhiB3 responds to O2 and nitric oxide via its [4Fe-4S] cluster and is essential for nutrient starvation survival. Proc. Natl. Acad. Sci. U.S.A. 104, 11562–11567 10.1073/pnas.0700490104 17609386 PMC1906726

[B117] Smeulders M. J. Keer J. Speight R. A. Williams H. D. (1999). Adaptation of *Mycobacterium smegmatis* to stationary phase. J. Bacteriol. 181, 270–283 9864340 10.1128/jb.181.1.270-283.1999 PMC103559

[B118] Smieja M. J. Marchetti C. A. Cook D. J. Smaill F. M. (2000). Isoniazid for preventing tuberculosis in non-HIV infected persons. Cochrane Database Syst. Rev. 2, CD001363 10796642 10.1002/14651858.CD001363 PMC6532737

[B119] Smith R. J. Schwartz E. J. (2008). Predicting the potential impact of a cytotoxic T-lymphocyte HIV vaccine: how often should you vaccinate and how strong should the vaccine be? Math. Biosci. 212, 180–187 10.1016/j.mbs.2008.02.001 18359048

[B120] Sousa E. H. S. Tuckerman J. R. Gonzalez G. Gilles-Gonzalez M. A. (2007). DosT and DevS are oxygen switched kinases in *Mycobacterium tuberculosis*. Protein Sci. 16, 1708–1719 10.1110/ps.072897707 17600145 PMC2203369

[B121] Stead W. W. Lofgren J. (1983). Does the risk of tuberculosis increase in old age? J. Infect. Dis. 147, 951–955 10.1093/infdis/147.5.951 6842028

[B122] Steyn A. J. Collins D. M. Hondalus M. K. Jacobs W. R. Kawakami R. P. Bloom B. R. (2002). *Mycobacterium tuberculosis* WhiB3 interacts with RpoV to affect host survival but is dispensable for in vivo growth. Proc. Natl. Acad. Sci. U.S.A. 99, 3147–3152 10.1073/pnas.052705399 11880648 PMC122487

[B123] Sud D. Bigbee C. Flynn J. L. Kirschner D. E. (2006). Contribution of CD8+ T cells to control of *Mycobacterium tuberculosis* infection. J. Immunol. 176, 4296–4314 16547267 10.4049/jimmunol.176.7.4296

[B124] Sun Z. Zhang Y. (1999). Spent culture supernatant of *Mycobacterium tuberculosis* H37Ra improves viability of aged cultures of this strain and allows small inocula to initiate growth. J. Bacteriol. 181, 7626–7628 10601224 10.1128/jb.181.24.7626-7628.1999 PMC94224

[B125] Taneja N. K. Dhingra S. Mittal A. Naresh M. Tyagi J. S. (2010). *Mycobacterium tuberculosis* transcriptional adaptation, growth arrest and dormancy phenotype development is triggered by vitamin C. PLoS ONE 5:e10860 10.1371/journal.pone.0010860 20523728 PMC2877710

[B126] Tews I. Findeisen F. Sinning I. Schultz A. Schultz J. E. Linder J. U. (2005). The structure of a pH-sensing mycobacterial adenylyl cyclase holoenzyme. Science 308, 1020 15890882 10.1126/science.1107642

[B127] Tufariello J. M. Jacobs W. R. Jr. Chan J. (2004). Individual *Mycobacterium tuberculosis* resuscitation-promoting factor homologues are dispensable for growth in vitro and in vivo. Infect. Immun. 72, 515–526 10.1128/IAI.72.1.515-526.2004 14688133 PMC343985

[B128] van der Wel N. Hava D. Houben D. Fluitsma D. van Zon M. Pierson J. (2007). *M. tuberculosis* and *M. leprae* translocate from the phagolysosome to the cytosol in myeloid cells. Cell 129, 1287–1298 10.1016/j.cell.2007.05.059 17604718

[B129] Voskuil M. Schnappinger D. Rutherford R. Liu Y. Schoolnik G. (2004a). Regulation of the *Mycobacterium tuberculosis* PE/PPE genes. Tuberculosis 84, 256–262 10.1016/j.tube.2003.12.014 15207495

[B130] Voskuil M. I. Visconti K. C. Schoolnik G. K. (2004b). *Mycobacterium tuberculosis* gene expression during adaptation to stationary phase and low-oxygen dormancy. Tuberculosis 84, 218–227 10.1016/j.tube.2003.12.014 15207491

[B131] Voskuil M. I. Schnappinger D. Visconti K. C. Harrell M. I. Dolganov G. M. Sherman D. R. (2003). Inhibition of respiration by nitric oxide induces a *Mycobacterium tuberculosis* dormancy program. J. Exp. Med. 198, 705–713 10.1084/jem.20030205 12953092 PMC2194188

[B132] Vynnycky E. Fine P. E. (1997). The natural history of tuberculosis: the implications of age-dependent risks of disease and the role of reinfection. Epidemiol. Infect. 119, 183–201 10.1017/S0950268897007917 9363017 PMC2808840

[B133] Walburger A. Koul A. Ferrari G. Nguyen L. Prescianotto-Baschong C. Huygen K. (2004). Protein kinase G from pathogenic mycobacteria promotes survival within macrophages. Science 304, 1800 15155913 10.1126/science.1099384

[B134] Wang C. C. Zhu B. Fan X. Gicquel B. Zhang Y. (2013). Systems approach to tuberculosis vaccine development. Respirology 18, 412–420 10.1111/resp.12052 23331331

[B135] Wang X. Wang H. Xie J. (2011). Genes and regulatory networks involved in persistence of *Mycobacterium tuberculosis*. Sci. China Life Sci. 54, 300–310 10.1007/s11427-011-4134-5 21267668

[B136] Wayne L. Lin K. (1982). Glyoxylate metabolism and adaptation of *Mycobacterium tuberculosis* to survival under anaerobic conditions. Infect. Immun. 37, 1042–1049 6813266 10.1128/iai.37.3.1042-1049.1982 PMC347645

[B137] Wayne L. G. (1977). Synchronized replication of *Mycobacterium tuberculosis*. Infect. Immun. 17, 528–530 409675 10.1128/iai.17.3.528-530.1977 PMC421156

[B138] Wayne L. G. Hayes L. G. (1996). An in vitro model for sequential study of shift down of *Mycobacterium tuberculosis* through two stages of nonreplicating persistence. Infect. Immun. 64, 2062–2069 8675308 10.1128/iai.64.6.2062-2069.1996 PMC174037

[B139] Wayne L. G. Sohaskey C. D. (2001). Nonreplicating persistence of *Mycobacterium tuberculosis*. Annu. Rev. Microbiol. 55, 139–163 10.1146/annurev.micro.55.1.139 11544352

[B140] Wigginton J. E. Kirschner D. (2001). A model to predict cell-mediated immune regulatory mechanisms during human infection with *Mycobacterium tuberculosis*. J. Immunol. 166, 1951–1967 11160244 10.4049/jimmunol.166.3.1951

[B141] Yu X. Xie J. (2012). Roles and underlying mechanisms of ESAT-6 in the context of *Mycobacterium tuberculosis*–host interaction from a systems biology perspective. Cell. Signal. 24, 1841–1846 10.1016/j.cellsig.2012.05.014 22634089

[B142] Zhang Y. (2004). Persistent and dormant tubercle bacilli and latent tuberculosis. Front. Biosci. 9:1136–1156 10.2741/1492 14977534

[B143] Zheng L.-L. Li Y.-X. Ding J. Guo X.-K. Feng K.-Y. Wang Y.-J. (2012). A comparison of computational methods for identifying virulence factors. PLoS ONE 7:e42517 10.1371/journal.pone.0042517 22880014 PMC3411817

